# Correlation between E-cadherin and p120 expression in invasive ductal breast cancer with a lobular component and MRI findings

**DOI:** 10.1007/s00428-017-2203-2

**Published:** 2017-08-04

**Authors:** Mary-Ann El Sharouni, Emily L. Postma, Paul J. van Diest

**Affiliations:** 1 Department of Pathology, University Medical Centre Utrecht, PO Box 85500, 3508 GA Utrecht, The Netherlands; 2 Department of Surgery, University Medical Centre Utrecht, PO Box 85500, 3508 GA Utrecht, The Netherlands; 3 Department of Pathology, University Medical Centre Utrecht, Heidelberglaan 100, 3584 CX Utrecht, The Netherlands

**Keywords:** Breast neoplasms, Magnetic resonance imaging, Biomarkers, Lobular

## Abstract

Invasive breast cancer comprises a spectrum of histological changes with purely lobular cancer on one side and purely ductal cancer on the other, with many mixed lesions in between. In a previous study, we showed that in patients with any percentage lobular component at core needle biopsy, preoperative MRI leads to the detection of clinically relevant additional findings in a substantial percentage of patients, irrespective of the percentage of the lobular component. Detection of a small lobular component may however not be reproducible among pathologists. Loss of membrane expression of E-cadherin or p120 is useful biomarkers of ILC and may therefore support a more objective diagnosis. All patients diagnosed with breast cancer containing a lobular component of any percentage between January 2008 and October 2012 were prospectively offered preoperative MRI. Clinically relevant additional findings on MRI were verified by pathology evaluation. Expression patterns of E-cadherin and p120 were evaluated by immunohistochemistry on the core needle biopsy. MRI was performed in 109 patients. The percentage of lobular component was significantly increased in cases with aberrant E-cadherin or p120 expression (both *p* = <0.001). However, aberrant expression of E-cadherin and p120 was not related to the probability of detecting relevant additional MRI findings. E-cadherin and p120 did not appear to be useful objective biomarkers for predicting additional relevant findings on MRI in patients with a lobular component in the core needle of their breast cancer.

## Introduction

Invasive breast cancer comprises a wide spectrum of specific histological types with varying clinical presentation, imaging characteristics, behavior and response to treatment. The histological spectrum of invasive breast cancers is a biological continuum with gradual transitions between the different types, e.g., invasive carcinomas with both ductal and lobular histological features frequently occur (“ductolobular,” IDLC). Invasive lobular carcinoma (ILC) often has a diffuse infiltrative growth pattern without microcalcifications and has a tendency to present in both breasts. Because of this, the extent of the lesion is frequently underestimated by mammography or ultrasound, increasing the risk of positive margins at breast surgery [1–7]. MRI has shown to be of added value for estimation of the extent of the lesion in ILC [2, 8, 9]. Patients with a core biopsy diagnosis of ILC therefore routinely undergo preoperative magnetic resonance imaging (MRI).

Recently, we showed that preoperative MRI in patients with IDLC yields clinically relevant additional finding in a substantial number of patients, irrespective of the percentage of the lobular component [9]. Since detecting a small lobular component may not be reproducible between pathologists, we searched for more objective biomarkers to determine if a preoperative MRI may be indicated in this selected patient category.

E-cadherin is the main cell-to-cell-adhesion molecule in the adherens junction of epithelial cells and its expression is very often lost in ILC by somatic mutations, deletions or promoter methylation. Because of this E-cadherin expression is a useful marker in the distinction between ILC and infiltrating ductal cancer (IDC) [10–12]. However, since only one allele may be affected with only partial loss of membrane expression, interpretation may be challenging in some cases. In literature, p120 has been suggested as another diagnostic marker in the distinction between ILC and IDC [13]. p120 is a catenin and also an essential component of adherens junctions. It is thought to be a key regulator of E-cadherin function, stability, and strength by directly influencing the adhesive strength by controlling the amount of cadherin available at the cell surface for adhesion [14–16]. Catenins respond differently to functional loss of E-cadherin. In ILC, p120 accumulates in the cytoplasm, whereas in IDC it is located on the membrane [13, 17, 18]. p120 may therefore be regarded as a useful diagnostic biomarker for lobular differentiation, next to E-cadherin.

We therefore set out to determine the value of E-cadherin and p120 expression patterns as potential objective biomarkers for predicting additional relevant findings on preoperative MRI in patients with invasive breast cancer with a lobular component of any size on core needle biopsy.

## Materials and methods

Between January 2008 and October 2012, all patients diagnosed with an invasive breast carcinoma showing lobular features on core needle biopsy were offered preoperative MRI as previously described [9]. Radiology reports of conventional imaging and MRI were compared and additional findings detected on MRI were registered as previously described [9].

All core needle biopsies were neutral buffered formaldehyde fixed, processed and paraffin embedded according to standard protocols. A dedicated breast cancer pathologist (PJvD) reviewed all slides and the percentage of lobular component was determined as previously described [9]. Surgical breast specimens were routinely processed.

**Fig. 1:**
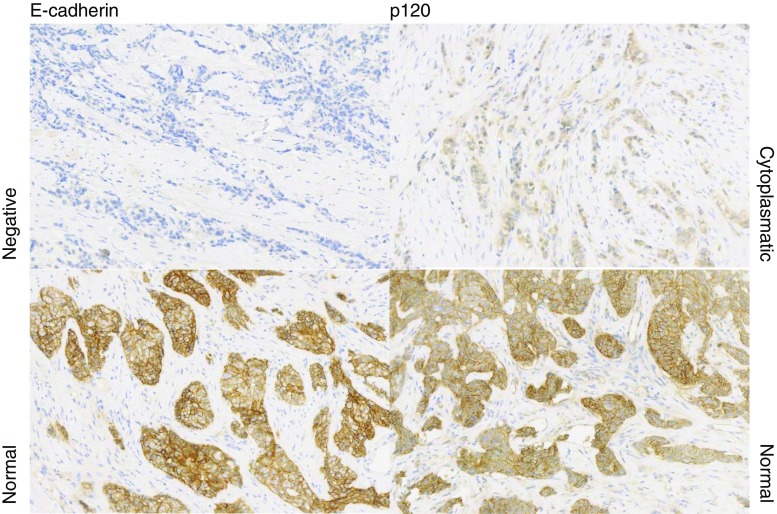
Examples of normal and aberrant staining for E-cadherin and p120 all at 10× magnification

E-cadherin and p120 were stained by immunohistochemistry on an automated immunostainer (BondMax, Leica) according to the manufacturer’s instructions. An experienced observer (PJvD) scored E-cadherin membrane staining as normal, reduced or negative, and p120 was scored as normal (membrane staining comparable to normal breast tissue), reduced or aberrant (cytoplasmic/negative) staining. Figure 1 shows examples of normal and aberrant staining for E-cadherin and p120.

### Statistical analysis

Data were analyzed using SPSS version 17.0. Normally distributed continuous variables were presented as means (standard deviation) and compared with independent *t* test. Not-normally distributed data were presented as medians (range) and compared with the Mann-Whitney U test. The chi-square test was used to compare percentages. A *p*-value <0.05 was considered significant. Patients were arbitrarily stratified into groups with different percentages of the lobular component; 0–30%, 31–70%, and 71–100% as before [9]. The Cochran-Armitage test was used to test for trend. Reduced E-cadherin and p120 staining was grouped with normal staining.

## Results

### Patients and imaging

Of the 505 patients diagnosed with invasive breast carcinoma, lobular differentiation was reported in 155 (31%) patients, ranging from 1 to 100% of lobular component at core needle biopsy. Patients whose MR-images were available (*n* = 109) were included in this analysis. The remaining 46 patients had either not undergone for MRI because of claustrophobia and obesity or had inadequate MR-imaging. All included patients were women with a mean age of 57.5 years. Seventy-two percent of the tumors were palpable. For a full table with baseline characteristics, we refer to our previous paper [9].

### Additional foci on MRI

As previously described, the quality of the slide was too low in one patient to be assessed for percentage of lobular component. Preoperative MRI detected additional malignant (“relevant MRI”) finding in 47/108 (44%) patients, 28/47 (60%) with additional ipsi- or contralateral foci, and more extensive disease (*≥*5.0 mm) in 19/47 (40%) patients [9]. Paraffin blocks in 99/109 (91%) had sufficient residual invasive cancer to perform E-cadherin and p120 staining.

### E-cadherin and p120 and lobular component

**Fig. 2:**
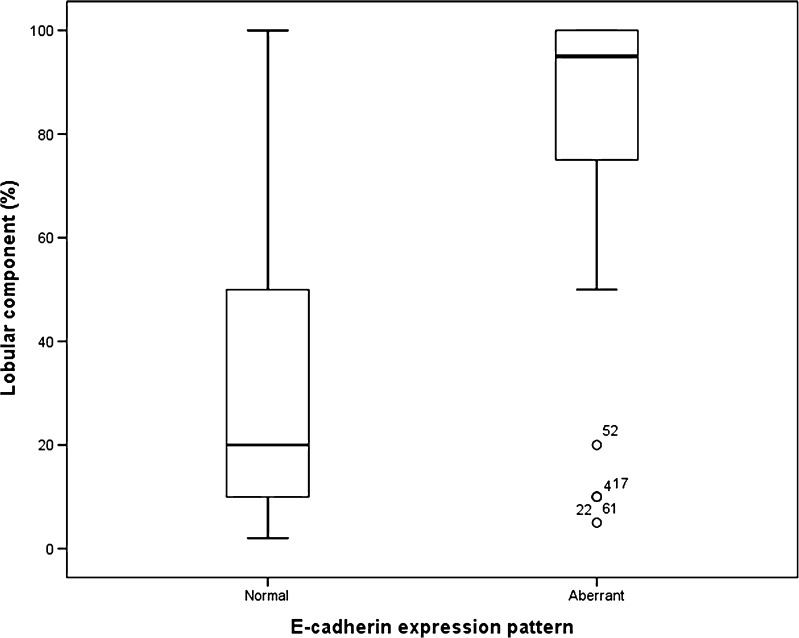
Lobular component in relation to E-cadherin expression pattern in 109 patients with ductolobular breast cancer (*p* = <0.001)

**Fig. 3:**
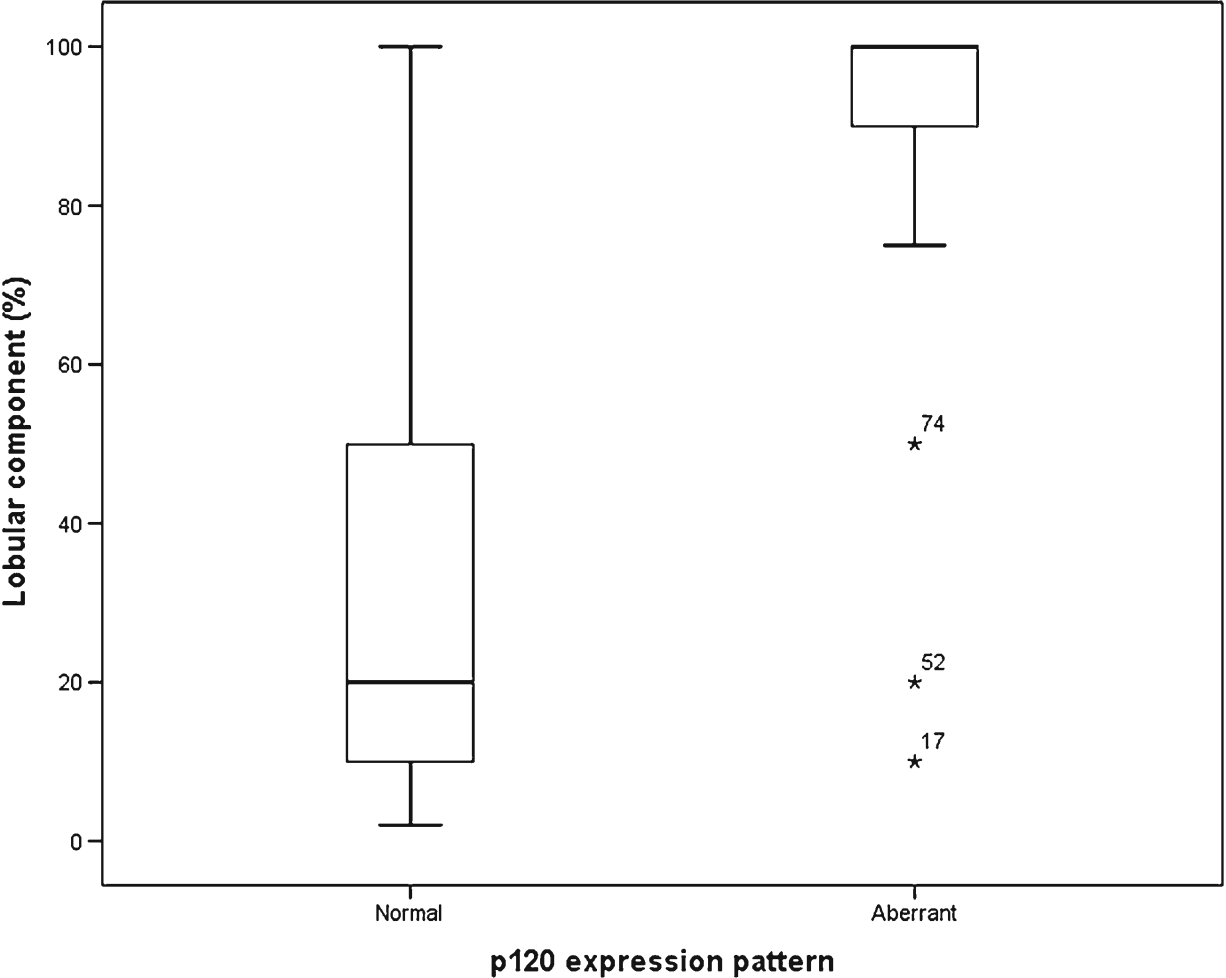
Lobular component in relation to p120 expression pattern in 109 patients with ductolobular breast cancer (*p* = <0.001)

E-cadherin was aberrant in 39/109 (39%) and p120 was aberrant in 29/109 (35%) of cases. The percentage lobular component was significantly higher in E-cadherin aberrant cases than in E-cadherin normal/reduced cases (*p* = <0.001, Fig. 2). Likewise, the percentage lobular component was significantly higher in p120 aberrant cases than in p120 normal/reduced cases (*p* = <0.001, Fig. 3). As shown in Table 1, neither E-cadherin nor p120 status could predict additional relevant findings in the preoperative MRI in the study population, also not when stratified for the percentage of the lobular component (100 vs. <100% in Table 2 1–30% vs. 31–70% vs. 71–100% in Table 3). Similarly, when subdividing the group of additional relevant findings into the group with patients with additional malignant foci and the group with more extensive disease, neither E-cadherin nor p120 status could predict additional relevant findings in the preoperative MRI (data not shown). Grouping the cases with reduced staining (7 for p120, 1 for E-cadherin) with normal or aberrant staining did not influence the results (data not shown).

**Table 1:** Probability of detecting clinically relevant additional MRI findings in breast cancer patients with any percentage lobular differentiation on core needle biopsy stratified for E-cadherin and p120 status

Additional MRI findings	E-cadherin expression		p120 expression	
	Normal	Aberrant	Normal	Aberrant
Relevant	24 (40%)	19 (49%)	29 (41%)	14 (48%)
None or not relevant	36 (60%)	20 (51%)	41 (59%)	15 (52%)
Total	60 (61%)	39 (39%)	70 (65%)	29 (35%)
	*p* = 0.258		*p* = 0.342

**Table 2:** Probability of detecting clinically relevant additional MRI findings in breast cancer patients with varying percentage of lobular differentiation on core needle biopsy, stratified for E-cadherin and p120 status

% lobular component	E-cadherin expression		p120 expression	
	Normal	Aberrant	Normal	Aberrant
1–99%				
Relevant	23 (41%)	11 (50%)	26 (41%)	8 (57%)
None or not relevant	33 (59%)	11 (50%)	38 (59%)	6 (43%)
	*p = 0.321*		*p = 0.202*	
100%				
Relevant	1 (25%)	7 (44%)	2 (40%)	6 (40%)
None or not relevant	3 (75%)	9 (56%)	3 (60%)	9 (60%)
	*p = 0.465*		*p = 0.704*

**Table 3:** Probability of detecting clinically relevant additional MRI findings in breast cancer patients with varying percentages lobular differentiation on core needle biopsy, stratified for E-cadherin and p120 status

% lobular component	E-cadherin expression		p120 expression	
	Normal	Aberrant	Normal	Aberrant
1–30%				
Relevant	14 (38%)	3 (60%)	15 (38%)	2 (100%)
None or not relevant	23 (62%)	2 (40%)	25 (62%)	0 (0%)
	*p = 0.317*		*p = 0.158*	
31–70%				
Relevant	5 (50%)	0 (0%)	5 (42%)	0 (0%)
None or not relevant	5 (50%)	3 (100%)	7 (58%)	1 (100%)
	*p = 0.196*		*p = 0.615*	
71–100%				
Relevant	5 (38%)	15 (50%)	8 (47%)	12 (46%)
None or not relevant	8 (62%)	15 (50%)	9 (53%)	14 (54%)
	*p = 0.360* *CA = p = 0.942*		*p = 0.600* *CA = 0.266*	

*CA* Cochrane armitage trend test.

## Discussion

In this study, we showed that E-cadherin and p120 do not appear to be useful objective biomarkers for defining presence of lobular component in a core needle biopsy in patients with IDLC. Nor does it predict for additional relevant findings on MRI. Patients with ILC routinely undergo MRI in order to better visualize the extent of the index lesion and identify contralateral lesions which occur at increased frequency. Ductolobular breast cancer has features of both ductal and lobular breast cancer, and biologically, one could expect an increased frequency of bilateral and/or ipsilateral additional foci in case of a smaller lobular component, as is the case for pure ILC. Indeed, we recently we showed for the first time that additional malignant MRI findings are found at the same frequency in ductal breast carcinoma with the smallest lobular component, starting from 2%, as in ductal breast carcinoma with a major lobular component [9].

The WHO defines mixed breast cancer subtypes as types that have “a specialized pattern in at least 50% of the tumor and a non-specialized pattern in between 10% and 49%.” [19]. Few studies consider IDLC a distinct type of breast cancer, but according to several studies, it is a carcinoma with mixed differentiation best to be compared with ILC [6, 7, 20]. A recent study by Arps et al. is perhaps the first article published considering ductal carcinoma with lobular component as a distinct type of breast cancer. They retrospectively selected 183 patients with IDLC breast cancer and quantified the lobular component into categories per 20%. The compared clinical and pathological data of these patients were compared to 1499 patients with ductal breast cancer and 375 patients with ILC. Their data confirm previous research and suggest that the clinical and biological characteristics of IDLC are more similar to that of ILC [21].

Diagnosing IDLC is however subjective and observer dependent, and thereby probably not optimally reproducible. In our study, in 24/109 patients, the lobular component was at revision classified as 100% at revision, suggesting these patients might not have been IDLC but ILC instead. This is within normal limits of inter-observer variability with regard to histologic typing in pathology, and conveniently allowed evaluation of MRI findings over the full spectrum from IDLC towards ILC. In order to find an objective biomarker for this selected group of patients, we investigated the expression of E-cadherin and p120, two proteins involved in adherens junction function which is often impaired in ILC [18, 22]*.* However, it should be noted that staining of E-cadherin and p120 is also dependent on technical details of the immunostaining protocol, which is why we used the normal surrounding breast parenchyma as internal control. Our results show that neither E-cadherin nor p120 is associated with additional malignant MRI findings in patients with IDLC, similar when stratifying for the percentage of the lobular component. Nevertheless, the percentage of the lobular component was significantly higher for cases with aberrant E-cadherin or p120 expression, consistent with previous research where both are biomarkers of lobular differentiation [13, 17, 18, 23]. About 40 and 30% of our IDLC cases had abnormal E-cadherin and p120 expression, respectively, confirming the in between nature of these cancers in the spectrum from pure IDC to pure ILC.

We agree that 80% of ILC showing aberrant E-cadherin expression is on the low side, but the usually higher numbers are based on resections rather than on biopsies as in the present study.

In conclusion, E-cadherin and p120 expression do not appear to be useful objective biomarkers for predicting additional relevant finding on MRI in patients with mixed ductal/lobular differentiation in core needle biopsies of their breast cancer.
